# Evaluation of apoptosis and angiogenesis in ectopic and eutopic stromal cells of patients with endometriosis compared to non-endometriotic controls

**DOI:** 10.1186/s12905-019-0865-4

**Published:** 2020-01-06

**Authors:** Ali-Akbar Delbandi, Mahmoud Mahmoudi, Adel Shervin, Sahel Heidari, Roya Kolahdouz-Mohammadi, Amir-Hassan Zarnani

**Affiliations:** 1 Immunology Research Center, Immunology and Infectious Disease Institute, Iran University of Medical Sciences, Tehran, Iran; 2 Immunology Research Center, School of Medicine, Mashhad University of Medical Sciences, Mashhad, Iran; 3 Reproductive Immunology Research Center, Avicenna Research Institute, ACECR, Tehran, Iran; 4 Department of Nutrition, School of Public Health, Iran University of Medical Sciences, Tehran, Iran; 5 Department of Immunology, School of Public Health, Tehran University of Medical Sciences, Tehran, Iran

**Keywords:** Endometriosis, Apoptosis, Angiogenesis, Stromal cells, Ectopic

## Abstract

**Background:**

Endometriosis is a chronic, painful, and inflammatory disease characterized by extra-uterine growth of endometrial tissues. Increased angiogenesis and resistance to apoptosis have been suggested to be involved in pathogenesis and development of endometriosis. The objective of this study was to examine apoptosis potential and angiogenesis contribution of eutopic (EuESCs) and ectopic (EESCs) endometrial stromal cells in patients with endometriosis compared to endometrial stromal cells from non-endometriotic controls (CESCs).

**Methods:**

Stromal cells were isolated by enzymatic digestion of ectopic (*n* = 11) and eutopic (*n* = 17) endometrial tissues from laparoscopically-confirmed endometriotic patients. Endometrial stromal cells of 15 non-endometriotic patients served as control. Following cell characterization by immunofluorescent staining and flow cytometry using a panel of antibodies, the total RNA was isolated from the cultured cells, and analyzed for the expression of genes involved in apoptosis (Bcl-2, Bcl-xL, Bax, and caspase-3) and angiogenesis [vascular endothelial growth factor-A (VEGF-A) and hepatocyte growth factor (HGF)] by Real-time PCR.

**Results:**

Significantly higher gene expression levels of Bcl-2 and Bcl-xL were found in EESCs compared with EuESCs and CESCs (*p* < 0.01). The gene expression of Bax in EESCs, EuESCs, and CESCs was not statistically significant. Furthermore, EuESCs exhibited a significantly lower caspase-3 gene expression compared with CESCs (*p* < 0.01) or EESCs (*p* < 0.05). Regarding angiogenesis, VEGF-A gene expression in EESCs (*p* < 0.001) and EuESCs (*p* < 0.05) were significantly higher compared with those of CESCs. EESCs exhibited a significantly higher HGF gene expression compared with EuESCs (*p <* 0.05).

**Conclusions:**

These findings suggest reduced propensity to apoptosis and increased angiogenesis potential of EESCs, which may be involved in pathogenesis of endometriosis.

## Background

Endometriosis, defined as settlement of endometrial glands and stroma in the extra-uterine cavity, is associated with irregular uterine bleeding, infertility, dyspareunia, and chronic pelvic pain [1]. The highest prevalence rates are typically found in couples with fertility problems (5–50%), while it affects 10–20% of reproductive-aged women [2]. Despite being quite common among women, our current understanding of the etiology and pathophysiology of endometriosis is unknown [3]. Several theories have been developed to address the pathogenesis of endometriosis, but the retrograde menstruation proposed by Sampson in 1927 is the most widely accepted one [4]. According to this theory, uterine endometrial cells refluxed through fallopian tubes into the peritoneal cavity during menstruation, implant and initiate the endometriotic lesion formation. Therefore, endometrial cells are likely to play an important role in the establishment of the disease [5].

Most recent reports have revealed an increased survival capability of ectopic (EESCs) and eutopic endometrial stromal cells (EuESCs) from patients with endometriosis compared with cells from non-endometriotic women [6]. Higher capacity for survival and proliferation, and resistance to apoptosis have been suggested to be involved in implantation of these cells in patients with endometriosis [6–8].

Apoptosis, maintains cellular homeostasis through elimination of excess or dysfunctional cells from the functional layer of the uterine endometrium during the late secretory and menstrual phases of the menstrual cycle [9]. Apoptotic activity of endometriotic cells is regulated by a diversity of regulatory factors. Among these regulators, both anti-apoptotic (e.g. B-cell lymphoma/leukemia-2 gene (Bcl-2) and B-cell lymphoma-extra large (Bcl-xL)) and pro-apoptotic factors (e.g. Bax and caspase-3) play a critical role in this process [9]. Several studies pointed that endometrium from endometriotic patients is less sensitive to apoptosis than that from healthy controls [10, 11].

Besides of apoptosis resistance, endometriotic lesions have been indicated to be highly vascularized with new vessels that are essential for the successful implantation of endometrial cells at ectopic sites [12]. The vascular endothelial growth factor-A (VEGF-A) is a potent angiogenic factor that induces endometrial cell proliferation and is considered an important factor in uterine angiogenesis [13]. Apart from VEGF-A, hepatocyte growth factor (HGF), as a pleiotropic cytokine, has angiogenic, mitogenic, and motogenic activities all of these may be involved in the pathogenesis of endometriosis [14].

According to these arguments, here we assessed expression of some genes actively involved in regulation of apoptosis (Bcl-2, Bcl-xL, Bax, and caspase-3) and angiogenesis (VEGF-A and HGF) in EuESCs, EESCs and endometrial stromal cells from non-endometriotic controls (CESCs).

## Materials and methods

### Patients

The study group included twenty-five women with ovarian endometriosis (mean age: 29 ± 7 years) and twenty with benign gynecological conditions (mean age: 32 ± 6 years). Endometriosis in patient groups was confirmed by laparoscopy and pathological examination. Participants in the control group did not exhibit any endometriotic lesions as carefully evidenced by a laparoscopic surgeon. All of the subjects were at the proliferative phase of the menstrual cycle with regular menstrual cycles and none of them were on or had received any hormonal or immunomodulatory treatment within 3 months before surgery. Patients with pelvic inflammatory disease, adenomyosis, and any malignancy or autoimmune disorders were excluded. All laparoscopic procedures were done by the same experienced gynecological surgeon. When endometriosis was diagnosed, the stage of the disease was determined according to the revised American Society for Reproductive Medicine classification system (ASRM 1996). Only women with endometrioma who were at stage III-IV were chosen for the study.

This study was approved by the Institutional Review Board and the Ethics Committee for Medical Research of the Avicenna Research Institute and all participants signed written informed consent before participating in the study.

### Sample collection

The ectopic endometrial tissues were taken through laparoscopy and eutopic samples were collected by biopsy curette. All of the endometrial samples were immediately transferred to the laboratory in tissue culture medium containing antibiotics, frozen in freezing medium containing Dulbecco’s modified Eagle’s medium (DMEM)-F12 (Sigma, USA), 10% fetal bovine serum (FBS; Sigma, USA), and 20% dimethyl sulfoxide (Sigma, USA) at − 80 °C and stored in liquid nitrogen until stromal cell isolation. A fraction of each tissue was sent for pathological confirmation of endometrioma.

From 25 endometriosis patients and 20 control patients, some samples were excluded from the study as a result of tissue contamination, inconsistent pathology report or absence of enough cell growth especially in case of EESCs. Finally, cells from 17 eutopic and 11 ectopic endometrial tissues of endometriotic patients and 15 eutopic endometrial tissue from non-endometriotic patients were used in this study.

### Isolation and culture of endometrial stromal cells (ESCs)

As previously described [15], ectopic and eutopic endometrial tissues of patients with ovarian endometriosis and eutopic endometrial tissues from control subjects were thawed and digested at 37 °C for 1.5–2 h in the presence of collagenase A and DNAse (Roche, USA). The obtained single cells were cultured, and non-adherent cells were removed by washing, and adherent cells were allowed to grow and reach to approximately 80% confluence. The identity and purity of the ESCs were evaluated by flow cytometry and immunofluorescent staining using a panel of antibodies against CD9, CD10, CD29, CD34, CD38, CD44, CD45, CD73, CD105, CD133 (all from Becton Dickinson Biosciences, USA), FITC-vimentin, FITC-cytokeratin (both from Abcam, USA), and PE-nestin (R&D Systems, USA) as described elsewhere [15].

### Quantitative real-time polymerase chain reaction

Total RNA of the ESCs was isolated according to the protocol supplied with RNA-Bee Reagent (BioSite, Sweden) according to the manufacturer’s instruction. The purity and concentration of RNA samples were determined using PicoDrop spectrophotometer (Picopetol, UK). One microgram of total RNA was reverse transcribed to complementary DNA (cDNA) according to the protocol published elsewhere [16]. The beta-actin gene was used as an internal control. Real-time PCR analyses were carried out with the SYBR green dye-based detection system (Takara, Japan) using the ABI 7500 thermocycler with fluorescence detection (Applied Biosystems, USA). Reactions were denaturated at 95 °C for 10 s, followed by 40 cycles of 95 °C for 5 s, 34 s of extension at 60 °C and finally a dissociation step consisting of 95 °C for 15 s, 60 °C for 1 min, 95 °C for 15 s, and 60 °C for 15 s. At the end of the program, a melting curve analysis was done and the PCR products were also analyzed using gel electrophoresis to ensure the accuracy of the amplifications. Reactions were done independently in triplicate. The primer sequences and the size of amplicons are shown in Table 1. The relative gene expression of apoptosis and angiogenesis genes were calculated by LinRegPCR.11.0 and REST software (REST– version 2009).

**Table 1:** Specifications of the primers used in this study

Target gene	Accession No.	Sequence 5′ to 3’	Amplicon size (bp)
Bcl-2-F	BC027258, NM_000633, NM_000657	ATCGCCCTGTGGATGACTGAGT	127
Bcl-2-R		GCCAGGAGAAATCAAACAGAGGC	
Bcl-xL-F	NM_001191, NM_138578	GCCACTTACCTGAATGACCACC	131
Bcl-xL-R		AACCAGCGGTTGAAGCGTTCCT	
Bax-F	NM_001291428, NM_001291429, NM_001291430, NM_001291431	TCAGGATGCGTCCACCAAGAAG	103
Bax-R		TGTGTCCACGGCGGCAATCATC	
Caspase-3-F	NM_004346, NM_032991	GGAAGCGAATCAATGGACTCTGG	146
Caspase-3-R		GCATCGACATCTGTACCAGACC	
VEGF-A-F	AF022375, NM_001025366, NM_001025367, NM_001025368	TTGCCTTGCTGCTCTACCTCCA	126
VEGF-A-R		GATGGCAGTAGCTGCGCTGATA	
HGF-F	NM_001010932.2, NM_000601.5	GCAATTAAAACATGCGCTGACA	140
HGF-R		TCCCAACGCTGACATGGAAT	
β-Actin-F	P60709	AGC CTC GCC TTT GCC GA	174
β-Actin-R		CTG GTG CCT GGG GCG	

### Statistical analysis

In this study we used REST software (version 2009) to test the group difference for significance using the Pair Wise Fixed Reallocation Randomization Test. Expression of each gene in each individual sample was first normalized to the corresponding housekeeping gene and then the ratio of normalized gene expression was compared between two groups. Data were expressed as mean ± standard error. A *p* < 0.05 was considered significant.

## Results

**Fig. 1:**
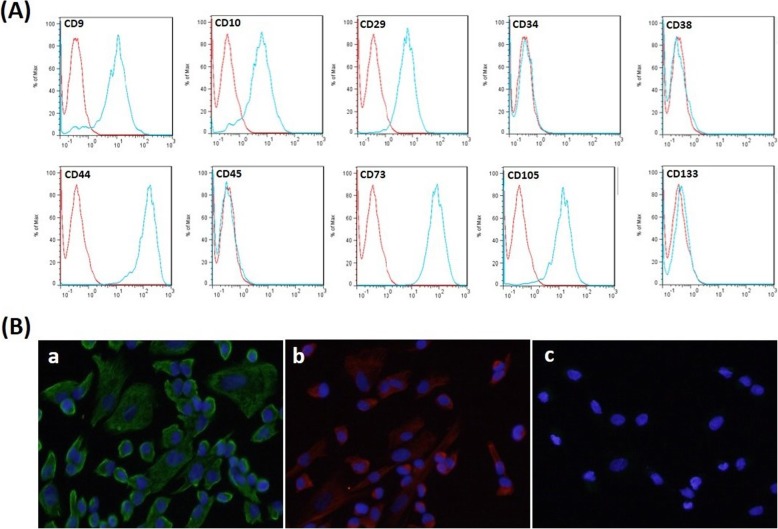
Immunophenotyping of ESCs of the study groups characterized by flow cytometric analysis and immunofluorescent staining. (**A**) Representative flow cytometric analysis of CD9, CD10, CD29, CD34, CD38, CD44, CD45, CD73, CD105, and CD133 markers in ESCs. **(B)** Representative immunofluorescent staining of ESCs exhibiting the expression of vimentin (a) and nestin (b) and negative immunoreactivity for cytokeratin (c). ESCs from all three sources exhibited the same pattern

ESCs exhibited a fibroblast-like appearance throughout the culture period. The flow cytometric analysis showed that isolated ESCs express mesenchymal origin antigens such as CD9, CD29, CD44, CD73, and CD105 but were negative for other origin markers, including CD34, CD38, CD133, and CD45 (Fig. 1A). Immunofluorescence staining of the propagated cells from all three sources showed positive results for vimentin, a stromal cell cytoskeletal marker, and nestin but negative signal for cytokeratin, an epithelial marker (Fig. 1B), suggesting purity of the isolated cells [15].

### Expression of apoptosis and angiogenesis related gene

**Fig. 2:**
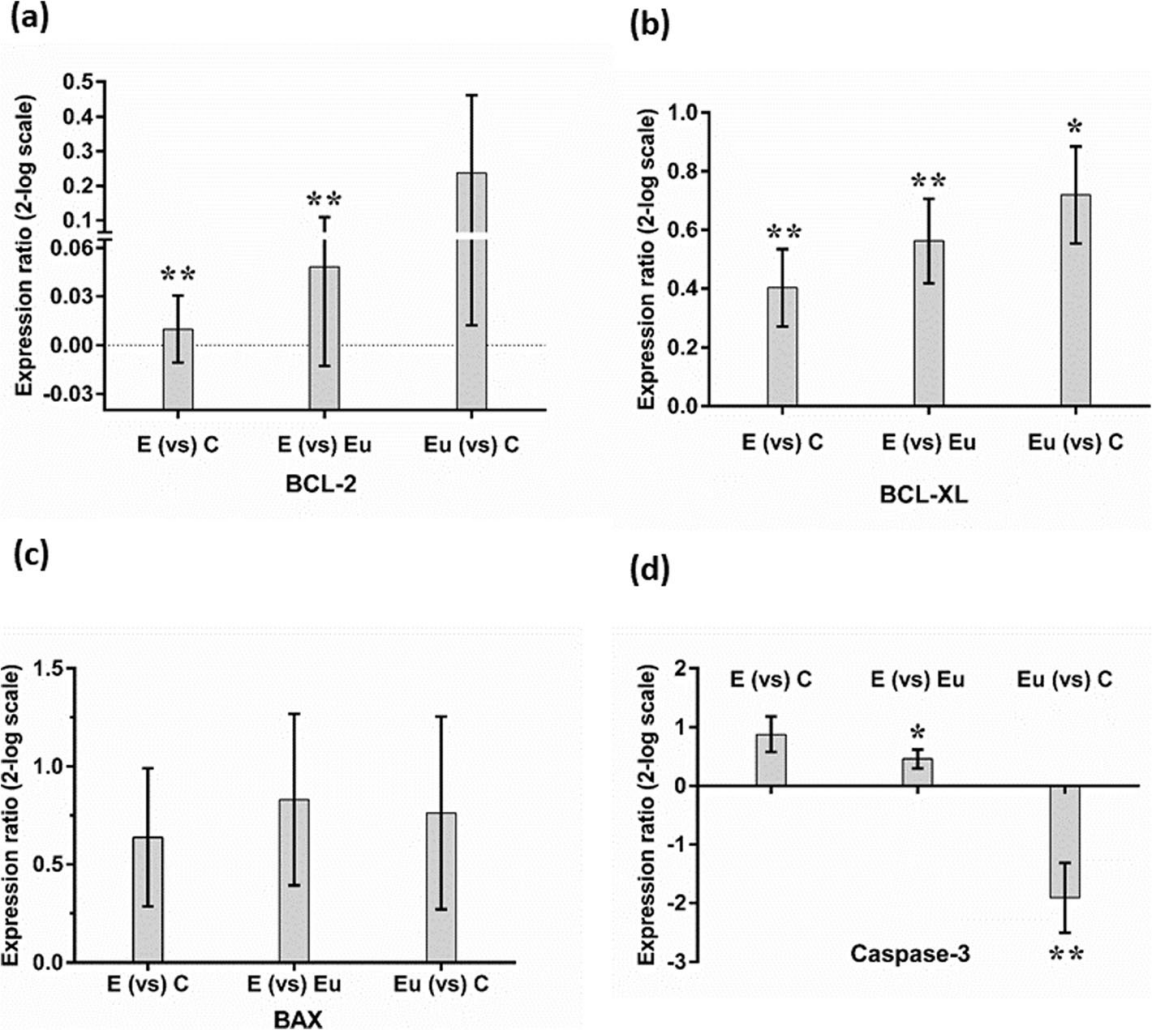
Bcl-2 (**a**), Bcl-xL (**b**), Bax (**c**), and caspase-3 (**d**) gene expression of isolated endometrial cells of studied groups. Each bar represents levels of Bcl-2, Bcl-xL, Bax, and caspase-3 gene expression in two different endometrial stromal cell groups. 17 Eu, 11 E and 15 C were used in this study. Data are expressed as mean ± standard error. E, stromal cells from the ectopic endometrium of patients with endometriosis (endometrioma); Eu, stromal cells from the eutopic endometrium of patients with endometriosis; C, stromal cells from non-endometriotic controls, *p < 0.05,** *p <* 0.01

**Fig. 3:**
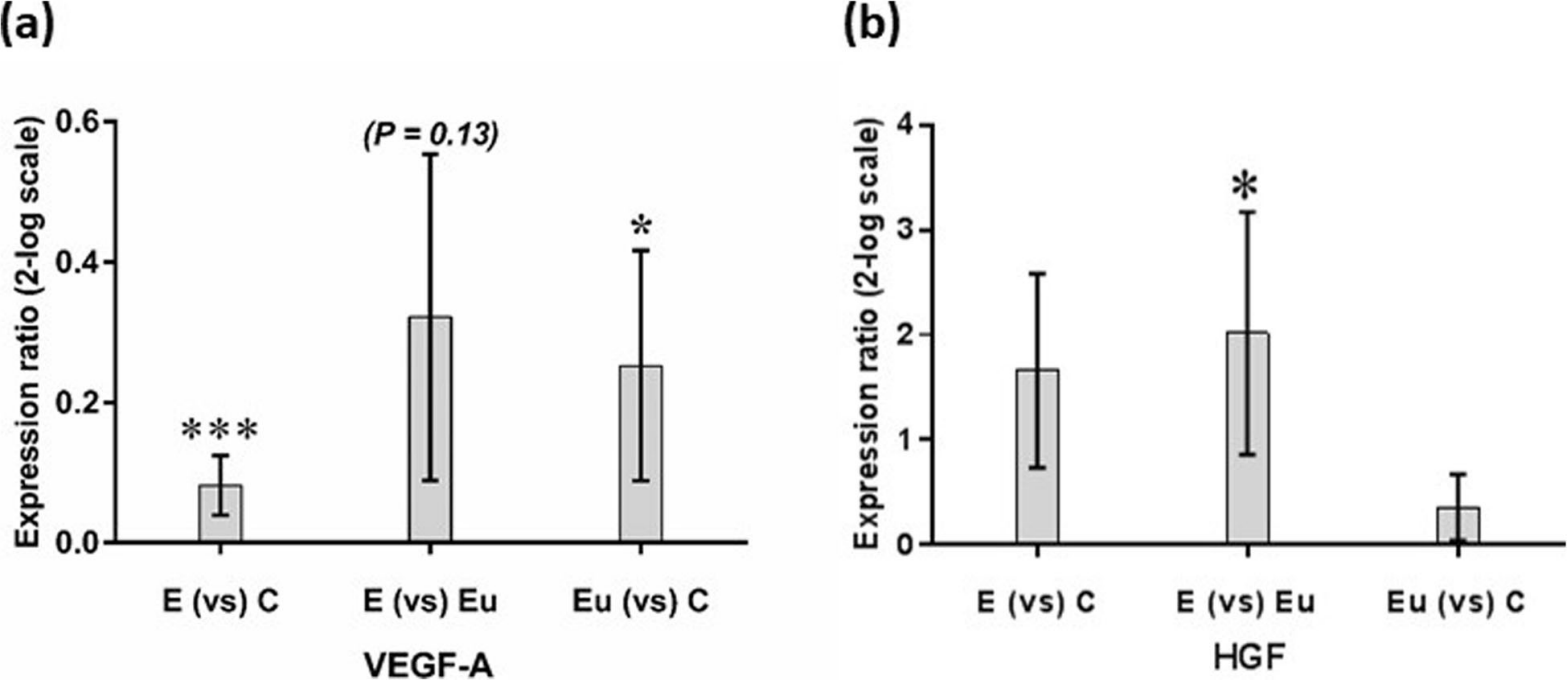
VEGF-A (**a**) and HGF (**b**) gene expression of isolated endometrial cells of studied groups. Each bar represents levels of VEGF-A and HGF gene expression in two different endometrial stromal cell groups. 17 Eu, 11 E and 15 C were used in this study. Data are expressed as mean ± standard error. E, stromal cells from the ectopic endometrium of patients with endometriosis (endometrioma); Eu, stromal cells from the eutopic endometrium of patients with endometriosis; C, stromal cells from non-endometriotic controls. *p < 0.05, *** p < 0.001

The gene expression levels of apoptosis and angiogenesis were examined using real time PCR. Our results showed significantly higher gene expression levels of Bcl-2 (Fig. 2a) and Bcl-xL (Fig. 2b) in EESCs compared to EuESCs or CESCs (*p* < 0.01). Besides, EuESCs showed elevated levels of Bcl-xL, but not Bcl-2, gene expression compared with CESCs (*p* < 0.05) (Fig. 2b and a respectively). The difference of Bax gene expression between EESCs, EuESCs, and CESCs was not statistically significant (Fig. 2c). In addition, EuESCs showed a significantly lower caspase-3 gene expression compared with CESCs (*p* < 0.01) or EESCs (*p* < 0.05), but the difference between EESCs and CESCs was not significant (Fig. 2d). VEGF-A gene expression by EESCs and EuESCs were statistically higher compared with those of CESCs (*p* < 0.001 and *p* < 0.05, respectively). Although EESCs showed increased VEGF-A gene expression compared to EuESCs, but the difference was not significant (Fig. 3a). HGF gene expression by EESCs was statistically higher compared with EuESCs (*p* < 0.05) (Fig. 3b).

## Discussion

Endometriosis is a benign disease characterized by the accumulation of cells in ectopic sites that could result from either increased angiogenesis or the deficiency of cells to undergo apoptosis [17].

Apoptosis is a form of programmed cell death that removes old layers of functional endometrial cells at the late menstruation secretory phase and in this way maintains cellular homeostasis in the menstrual cycle [18]. The results of our study showed significantly higher gene expression levels of anti-apoptotic proteins, Bcl-2 and Bcl-xL, in EESCs compared with EuESCs or CESCs. The pro-apoptotic caspase-3 gene revealed lower expression levels in EuESCs compared with CESCs or EESCs. In addition, the difference of Bax gene expression between ESCs from all three sources was not statistically significant. Extrinsic and intrinsic apoptotic pathways may be involved in the pathogenesis of endometriosis [19]. FasL/Fas interaction in extrinsic pathway leads to a cascade of activation of initiator (caspase-8 and -9) and effector

(caspase-3, − 6 and/or − 7) caspases, and finally apoptosis [19]. In a study by Nisio et al. caspase-8 but not caspases 9 and 3 overexpressed in cortical tissue surrounding endometriotic cysts and not cortex around other benign cysts. These findings suggested that altered cellular microenvironment could induce cellular damage of normal tissue around endometriotic cyst [20]. Besides, few studies have investigated Fas expression in endometriotic tissues. In a study by Harada et al., Fas showed random expression in both eutopic and ectopic endometrial tissues and authors suggested that Fas may be less involved as an apoptosis regulator in both eutopic and ectopic endometrial tissues [21]. On the other hand, increased levels of inflammatory cytokines [22], growth factors [23], and especially soluble/active FasL [24] have been demonstrated in peritoneal fluid (PF) of women with moderate to severe endometriosis compared to women with early-stage disease or disease-free women. Increased levels of soluble/active FasL in PF of endometriotic patients may contribute to increased apoptosis of Fas-bearing immune cells [24]. Furthermore, in studies by Garcia-Velasco et al. [25] and Selam et al. [26], increased levels of platelet-derived growth factor (PDGF), transforming growth factor-ß (TGF-ß), and IL-8 induced expression of FasL by endometriotic stromal cells. The authors speculated that elevated PF levels of these factors induces apoptosis of immune cells but not endometriotic tissues via stimulation of FasL/Fas interaction. So based on the above findings it seems that FasL/Fas mediated apoptosis pathway is not efficient in elimination of endometriotic implants. Nevertheless, further studies are needed to investigate FasL/Fas expression in endometrial and endometriotic tissues. So in this study we aimed to investigate gene expression of factors involved in intrinsic pathway of apoptosis.

Several gene expression studies related to apoptosis were done on human endometrial cells during the different phases of the menstrual cycle. These studies provided conflicting data regarding the difference in gene expression levels involved in apoptosis between normal endometrial tissue and endometrial tissue in women with endometriosis [7, 27]. Although a large number of studies have examined apoptosis through regulation of Bcl-2 expression in endometriosis and a relatively few studies have examined apoptosis through regulation of Bax expression in endometriosis, very few studies have monitored apoptosis through regulation of caspase-3 and Bcl-xL expression in endometriosis. With regard to apoptosis, many studies showed an inverse correlation between the level of apoptosis and the stage of the disease [11, 28]. The Bcl-2 protein is probably the best protein for investigating apoptosis. Previous studies have provided conflicting results regarding the difference in Bcl-2 expression between normal endometrial tissue and endometrial tissue in women with endometriosis. According to published studies, Bcl-2 was negative in most of the ectopic endometrial tissues from endometrioma [21, 29] and in another study, Bcl-2 expression was not different between endometrial tissues of endometriotic women and control counterparts [27, 30], but it was significantly expressed to a greater extent in stromal cells from ectopic tissues in another study [31]. On the other hand, in a study by Meresman et al., an increased expression of Bcl-2 protein was found in eutopic endometrium from women with endometriosis compared to the control group only in late proliferative phase [17]. Similarly to Jones et al. [31] study, in our study, Bcl-2 expression was increased significantly in EESCs.

Regarding Bcl-xL, gene expression of this anti-apoptotic protein was not different between women with endometriosis compared to healthy controls [32]. On the contrary, we found increased mRNA expression of Bcl-xL gene in EESCs compared with EuESCs and CESCs. Apart from anti-apoptotic proteins, pro-apoptotic proteins such as Bax and caspase-3 may have fundamental roles in pathogenesis of endometriosis. Bax gene is one of the well-characterized pro-apoptotic genes, antagonizes the prosurvival activity of Bcl-2 [33]. Goumenou et al., found a strong inverse correlation between Bax and Bcl-2 expression in endometrioma [34]. In our study, we observed no statistically significant differences between EESCs, EuESCs, and CESCs in terms of Bax gene expression. Similarly, in previous studies, the Bax gene expression did not differ between endometrium from women with endometriosis and controls [17, 27, 30, 32]. This finding may imply that not all genes involved in regulation of apoptosis are modulated in patients with endometriosis.

Caspase-3 is a member of the cysteine protease family, plays a fundamental role in the activation of apoptosis [35]. We studied caspase-3 gene expression in ESCs because of its important role in apoptosis but more so because in several of the studies related to endometriosis that we studied it was altered or lost. In our study caspase-3 gene expression was significantly lower in EuESCs compared to CESCs. Similarly, in a study by Wei et al., significantly lower expression of caspase-3 protein was found in ectopic and eutopic endometrium of patients with endometriosis as compared with the control group [36]. The discrepancy between results in different studies could be explained by: 1) the properties of separated cells (glandular versus stromal cells), 2) the use of different endometriotic lesions (peritoneal versus ovarian endometriosis), 3) various PCR techniques, 4) difference in the preparation of cell and tissue culture conditions, 5) difference in age and sexual phase of patients, and 6) difference in the stage of disease and in the number of subjects.

In general, with regard to above findings, increased gene expression of Bcl-2 and Bcl-xL as anti-apoptotic proteins in EESCs and decreased gene expression of caspase-3 in EuESCs, may hamper apoptosis and lead to abnormal cell growth in ectopic locations and the development of endometriosis.

Regardless of the apoptosis, angiogenesis has an essential role in the establishment and growth of endometriotic lesions. The highly regulated angiogenesis is responsible for maintaining normal reproduction and endometrial growth and remodeling [12]. VEGF-A as one of the most potent angiogenic factors, has a critical role in physiological and pathological angiogenesis [37]. VEGF-A production is stimulated by growth factors, hormones, cytokines, and hypoxia [38] and sources of this factor in endometriosis include ectopic endometrium and peritoneal macrophages [39]. Numerous studies have shown significantly increased VEGF levels in the PF of endometriotic patients compared to controls [40, 41]. Regarding VEGF-A gene expression, some studies showed unchanged [40, 42, 43], while other studies showed increased VEGF-A gene expression in eutopic or ectopic endometrium of women with endometriosis compared to endometrium of non-endometriotic women [44–48]. However, only one study investigated VEGF-A expression in ESCs and in that study, VEGF-A expression was not different between EuESCs and CESCs [49]. Our results showed the increased VEGF-A gene expression in EESCs and EuESCs compared to CESCs. Discrepancy in mentioned findings might be attributed to difference in phases of the menstrual cycle as in a study by Danastas et al. [44], VEGF-A gene expression was significantly higher during menstruation than other phases. Besides, type of endometriotic lesions affects VEGF-A gene expression as higher VEGF-A gene expression was observed in highly vascularized red peritoneal lesions than black ones [12]. Genetic also plays a fundamental role in this regard as some polymorphisms of the VEGF gene might be protective or destructive for endometriosis [50].

Another important angiogenic factor is HGF. Lipopolysaccharides (LPS), inflammatory cytokines, and prostaglandins stimulate HGF production in pelvic cavity of endometriotic patients [51] and the peritoneum and endometriotic stromal cells seem to be primary sources of HGF in endometriosis [51, 52]. Increased HGF expression has been shown in ectopic compared to eutopic endometrial tissue in patient with endometriosis [43] or non-endometriotic patients [53]. Regarding ESCs, one study showed increased HGF protein expression in EuESCs compared to CESCs [54] and in line with our findings, in a study by Arablou et al. HGF gene expression was higher in EESCs compared to EuESCs [55].

HGF and its receptor c-met were shown to enhance the degradation of extracellular matrix (ECM) and stimulate invasion of shed eutopic and ectopic endometrium via autocrine and paracrine pathways [56]. Besides, HGF has been shown to promote VEGF-A-driven angiogenesis [57]. So these findings imply that HGF may play a role in the pathogenesis of endometriosis.

## Conclusion

The present study demonstrated that not only less susceptibility of EESCs and EuESCs to apoptosis, but also increased angiogenesis of these cells may result in their continuing growth into ectopic locations and would be fundamental to the pathophysiology of endometriosis thus further investigations on apoptosis and angiogenesis of EESCs and EuESCs compared to control groups are needed to clarify the roles of these factors in the development of endometriosis.

## Data Availability

The datasets used and/or analyzed during the current study are available from the corresponding author on reasonable request.

## Abbreviations

ASRM: American Society for Reproductive Medicine
Bcl-2: B-cell lymphoma/leukemia-2
Bcl-xL: B-cell lymphoma-extra Large
cDNA: complementary DNA
CESCs: Control Endometrial Stromal Cells
DMEM-F12: Dulbecco’s Modified Eagle’s Medium-F12
ECM: Extracellular Matrix
EESCs: Ectopic Endometrial Stromal Cells
ESCs: Endometrial Stromal Cells
EuESCs: Eutopic Endometrial Stromal Cells
FBS: Fetal Bovine Serum
HGF: Hepatocyte Growth Factor
LPS: Lipopolysaccharides
PDGF: Platelet-Derived Growth Factor
PF: Peritoneal Fluid
TGF-ß: Transforming Growth Factor-ß
VEGF-A: Vascular Endothelial Growth Factor-A
